# An Improved Method of Grafting Ulcers

**Published:** 1892-02

**Authors:** 


### AN IMPROVED METHOD OF GRAFTING ULCERS.

Dr. Gill, in a letter to the *Lancet*, says that having had an exceptionally large number of chronic ulcers of the leg, which incapacitated the patients from work, and finally brought them into the infirmary, he tried the ordinary methods of grafting, but being disgusted with the very large number of total failures he experienced, he undertook various experiments, and at last adopted the following plan, which he distinctly disclaims as his own, but which consists in adopting and combining the ideas of several people. The success he obtained with this method was so marked that he thinks a large number of practitioners at home and abroad (in India especially, where he found all ulcers very intractable under ordinary treatment) will welcome it. Even when the ulcer is deep, with hard thickened edges and extending all around the limb, the method applies. This is to cleanse the surface well for two or three days with boracic fomentations, and then (contrary to what he was taught) slightly abrade the granulations, just sufficient to cause oozing, and apply the graft directly to the abraded surface, where it is held in position by a small pile made of half-inch squares of green protective, four or five squares being placed one on the top of the other. A graft is thus applied to every square inch of surface.

And now comes the most important thing of all, and which is an idea he received from a friend. This is to encircle the limb with a fold of carbolic gauze, which extends two or three inches above and below the ulcer, where it is attached to the sound skin by collodion. The ulcer is then thoroughly dredged with boracic powder through the gauze, and the whole is wrapped in a layer of boracic wet lint, which is kept thoroughly moist. As a rule, the dressing is not disturbed for three days, when the lint is removed, and the limb, well irrigated with boracic lotion, the grafts remaining perfectly secure under their heaps of protective, which again is kept in position by the gauze. The limb is then redusted with boracic powder, and done up in the wet lint, which is now changed daily. At the end of ten days the gauze and protective are removed, and each graft will be found as large as a sixpence, while those near the edges will have exercised a spermatic influence, and caused a rapid ingrowing of epithelium.

Since adopting the above plan, Dr. Gill says that he has never lost a single graft, though employed on most unfavorable surfaces—a very different result to the old way of covering the grafts with a large piece of protective which retained some exudations under it, and thus bathed the tender graft in a poisonous medium, with a result that 80 per cent. of them never "took."—*The Cincinnati Lancet-Clinic.*

